# Amyopathic dermatomyositis may be on the spectrum of autoinflammatory disease: A clinical review

**DOI:** 10.1515/rir-2024-0005

**Published:** 2024-03-31

**Authors:** Saika Sharmeen, Lisa Christopher-Stine, Joann N. Salvemini, Peter Gorevic, Richard Clark, Qingping Yao

**Affiliations:** Division of Rheumatology, Allergy and Immunology, Department of Medicine, Stony Brook University Renaissance School of Medicine, Stony Brook, NY, USA; Johns Hopkins Myositis Center, Johns Hopkins University, Baltimore, MD, USA; Department of Dermatology, Stony Brook University Renaissance School of Medicine, Stony Brook, NY, USA

**Keywords:** Autoinflammatory disease, dermatomyositis, amyopathic dermatomyositis, periodic fever syndrome, Yao syndrome

## Abstract

Systemic autoinflammatory diseases (SAIDs) are distinct from autoimmune diseases. The former primarily results from abnormal innate immune response and genetic testing is crucial for disease diagnosis. Similar cutaneous involvement is a main feature for both SAID and dermatomyositis (DM), so they can be confused with each other. A literature search of PubMed and MEDLINE was conducted for relevant articles. The similarities and differences between these two types of diseases were analyzed. We found phenotypic similarities between these two types of disorders. Accumulating data supports a major role of the innate immune system and a similar cytokine profile. Molecular testing using an autoinflammatory disease gene panel may help identify SAID patients from the DM population and may offer therapeutic benefit using interleukin-1 (IL-1) inhibitors. A subset of DM, notably amyopathic dermatomyositis in the absence of autoantibodies may be on the spectrum of autoinflammatory disease.

## Introduction

Systemic autoinflammatory diseases (SAIDs) are an expanding group of rheumatic and inflammatory diseases primarily associated with abnormal innate immune responses, causing periodic fevers and inflammation involving multiple organs such as the skin.^[1]^ Since the introduction of the concept by a group of National Institutes of Health (NIH) physicians/researchers in 1999, this terminology has been widely used to encompass syndromes linked to genetic mutations. Generally, autoantibodies and antigen specific T cells are not detectable in systemic SAIDs.^[2]^ Since the completion of the Human Genome Project in 2003, advanced molecular technologies, such as next generation and whole exome/ genome sequencing have revolutionized disease discovery and mechanistic study of existing diseases. Hundreds of mutations have been identified for SAIDs and used for the disease diagnosis. As a result, this category of diseases has expanded rapidly. Prototypic diseases are hereditary periodic fever syndromes, such as Familial Mediterranean fever (FMF, OMIM#249100), Cryopyrin-associated periodic syndrome (CAPS, OMIM# 606416), TNF receptor associated periodic syndrome (TRAPS, OMIM # 142680), Hyper IgD syndrome (HIDS, OMIM#260920), and NLRP12 autoinflammatory disease (OMIM#611762); all are autosomal recessive or dominant disorders.^[3]^ SAIDs also include Nucleotide-binding oligomerization domain containing protein 2 (NOD2)-associated diseases like Blau syndrome (OMIM# 186580), Crohn’s disease and Yao syndrome (YAOS, OMIM# 617321).^[3]^

Dermatomyositis (DM) is an inflammatory disease affecting the skin and muscles; amyopathic dermatomyositis (ADM) is a subtype of this disorder in which muscle disease is minimal, intermittent or absent.^[4]^ Approximately 20% of DM patients are amyopathic.^[5]^ Characteristic features of DM are proximal muscle weakness along with pathognomonic cutaneous findings of Heliotrope eruption and Gottron’s papules/sign. Other characteristic polymorphous cutaneous findings are also reported.^[6]^ Internal organs, such as the lungs, can be involved in both DM and ADM. For example, ADM has been associated with rapidly progressive interstitial lung disease (RP-ILD).^[7]^ To date, treatment of this subgroup of ADM patients has been challenging due to their poor response to commonly used immunosuppressive agents.

Currently, DM is categorized among the systemic autoimmune diseases, and cutaneous presentation is a chief feature. Similarly, patients with SAIDs often present with skin rashes that can mimic DM, particularly ADM. While myositis specific antibodies (MSAs) are helpful for the diagnosis of DM, their positive rates are low, ranging from 20% to 50%. ^[8]^ It is, therefore, reasonable to assume that patients with SAIDs could be clinically confused or incorrectly diagnosed with DM, especially in the absence of MSAs. The distinction between these two entities is clinically important since their therapeutic strategies are different. In this review, we examine and analyze the similarities and differences between DM and SAIDs.

## Methods

We conducted a literature search of PubMed and MEDLINE between January 2000 and February 2023 using the key terms “dermatomyositis”, “amyopathic dermatomyositis”, “polymyositis”, “autoinflammatory disease”, periodic fever syndrome”, “genetics”, “biologic”, and “IL-1 inhibitor”. Pertinent publications in English were reviewed and data obtained to compare clinical and pathogenic similarities and differences between DM and SAIDs.

## Results

### Cutaneous Manifestation Associated with DM

Patients with DM or ADM characteristically present with pink-violaceous erythema of the eyelids, with or without edema (Heliotrope eruption), pink-violaceous papules overlying the interphalangeal and metacarpophalangeal joints (Gottron’s papules) and macular pink-violaceous erythema overlying joints, such as the elbow, knee, and ankle (Gottron’s sign). A fine scaly pink-violaceous discoloration may occur on the neck (V sign), hips and thighs (Holster sign), upper back and shoulders (Shawl sign) and scalp with or without hair loss. Nail fold changes include periungual erythema, atrophy, and telangiectasia.^[9]^ Common cutaneous symptoms include pruritus and photosensitivity, which may impact quality of life and can even be debilitating. ADM can be associated with positive or negative autoantibodies. MSAs are helpful in the diagnosis, outcome prediction, and management of DM. DM-specific antibodies include anti-Mi2, anti–MDA5, anti-NXP2, anti-TIF1-β, and anti-small ubiquitin-like modifier activating enzyme (SAE) among others^[10]^ (Table 1). MDA5 autoantibodies are often associated with ADM and predict a poor prognosis due to RP-ILD.^[11]^ Anti-TIF1 γ antibodies are detected in ADM and associated with distinct skin features and malignancy.^[12]^ Positive rates of MSAs (MDA-5 and TIF1-y antibodies) in ADM are low ranging from 13% to 26%.^[12]^ Approximately 70% of ADM patients do not have detectable autoantibodies. Therefore, it is worthwhile exploring a potential role of autoinflammation in this subgroup of DM patients.

**Table 1:** Myositis specific autoantibodies and their association with clinical phenotypes

Autoantibody	Clinical implications
Mi-2	Characteristic dermatomyositis skin lesions: V- and Shawl signs
	Better prognosis
SRP	Necrotizing myopathy
	Refractory to treatment
Anti-synthetase	Fever, inflammatory arthritis, polymyositis, ILD, and mechanic’s hand
	Extra muscular involvement may be initial presentation. PL7 and PL12 more severe lung disease without muscle involvement
MDA-5	Amyopathic dermatomyositis
	Arthritis
	Rapidly progressive ILD
	Pneumomediastinum
	Skin erosions, tender gums, palmar papules, cutaneous ulceration
NXP2	Skin calcinosis
	Subcutaneous edema
	Malignancy - adult
	Distal muscle weakness, neck weakness, gastrointestinal bleeding-perforation, lower risk of ILD
TIF 1 γ(p155)	Malignancy
	Ovoid palatal patch
	Psoriatic like plaques (red on white)

Dermatomyositis (DM), Anti-melanoma differentiation-associated protein 5 (MDA5), Anti-signal recognition particle (SRP)), Myositis-associated autoantibodies, Anti-nuclear matrix protein 2 antibody (NXP2), Anti-transcription intermediary factor 1 (TIF1)-γ, Interstitial Lung Disease (ILD)

### Clinical Manifestation Associated with SAIDs

Patients with autoinflammatory diseases commonly present with a complete or partial constellation of recurrent fever, rash, arthralgias, myalgias, chest pain, pleuritis, pericarditis, and abdominal pain/diarrhea. These presentations are called autoinflammatory phenotypes in the field of SAIDs. Patients frequently undergo thorough evaluation with negative serologic profiles for antinuclear antibodies (ANA), anti-extractable antigen (anti-ENA), anti-double stranded DNA, and antineutrophil cytoplasmic antibodies (ANCA). Gastrointestinal evaluations including esophagoduodenoscopy and colonoscopy with pathologies are negative for inflammatory bowel disease.^[13]^ For a correct diagnosis of these SAIDs, molecular testing is often performed using an autoinflammatory disease or periodic fever syndrome panel to search for genetic mutations or variants.^[14]^

### Comparison of Cutaneous Presentations of SAIDs with DM

**Figure 1:**
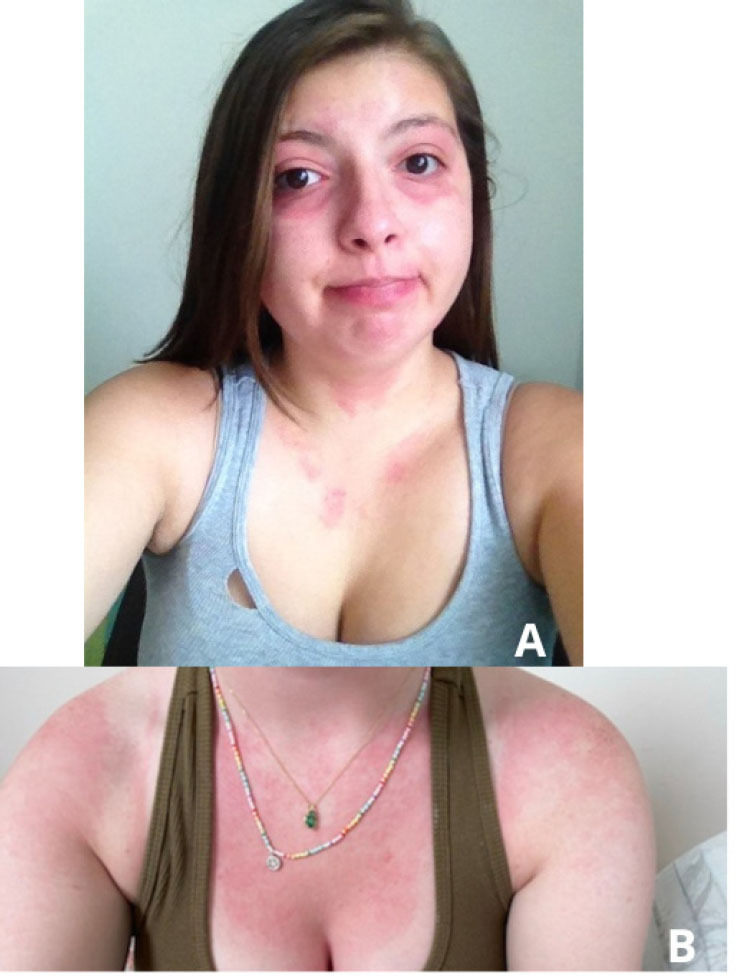
Periorbital purple rash and rash on anterior neck and chest in a TRAPS patient with carriage of heterozygousTNFRSF1A R92Q mutation (A). Diffuse redness on upper chest and shoulders mimicking “V” sign in a patient with NLRP12-autoinflammatory disease, who carries the NLRP12 mutation, F402L (B).

Common to both SAIDs and DM are prominent cutaneous features. Due to the shared clinical phenotypes, SAIDs should be considered as one of the differential diagnoses for DM particularly in the absence of autoantibodies. Skin manifestations vary in different SAIDs, including urticarial or maculopapular rashes, neutrophilic dermatosis, erythematous patches, among others (Table 2).^[15, 16, 17, 18, 19, 20, 21]^ Skin presentations associated with SAIDs can be nonspecific ^[22]^ and mimic those associated with DM/ADM (Figure 1),^[23]^ some of which could be interpreted as Heliotrope rash, Gottron’s sign, or V sign. The skin has limited responsiveness and therefore diagnosis cannot rely on eruptions only; close correlation between clinical, pathologic and, at times, laboratory studies are required to arrive at a correct diagnosis. The classic histopathology in DM includes a vacuolar interface dermatitis with epidermal atrophy and hyperkeratosis with mucin deposition in the dermis.^[8]^ However, nonspecific dermatopathological findings can also be seen.^[24]^ Given the above, it seems likely that molecular analysis of genetic markers for SAIDs may help identify SAID patients from the DM patient population, particularly in the absence of autoantibodies.

**Table 2:** Characteristic skin phenotypes and dermatopathological findings of SAIDs

SAID	Gene	Skin	Pathology
FMF	MEFV	Erysipelas-like erythema, often on the lower extremities with tissue swelling.	Edema of the superficial dermis and sparse perivascular infiltrates with lymphocytes, neutrophils, histiocytes, and nuclear dust. IgM, C3, and fibrinogen in the capillary walls of the papillary dermis.
CAPS or NLRP3-AID	NLRP3	Urticarial rashes.	Neutrophilic urticarial dermatosis with a perivascular and neutrophilic infiltrate with limited leukocytoclasia.
TRAPS	TNFRSF1A	Various rash. Maculopapular, edematous patches, erysipelaslike or urticarial rashes.	Perivascular and interstitial lymphocytic and monocytic infiltrate.
HIDS or MD	MVK	Maculopapular or morbilliform rashes, urticarial.	Nonspecific findings, perivascular inflammatory infiltrate of polymorphonuclear neutrophils and few lymphocytes.
NLRP12-AID	NLRP12	Urticarial rashes.	No data available.
Yao syndrome	NOD2 variant IVS8+158 in nearly all and concurrent R702W or other variants.	Erythematous patches or plaques mostly, urticaria-like, livedo reticularis occasionally.	Spongiotic dermatitis mostly.
Blau syndrome	NOD2 mutations on exon 4	Maculopapular, nodular.	Non-caseating, sarcoid-type granulomas.

Note: SAID, systemic autoinflammatory disease; FMF, Familial Mediterranean Fever; CAPS, Cryopyrin-associated periodic syndrome; TRAPS, tumor necrosis factor receptor-associated periodic syndrome; HIDS, Hyperimmunoglobulinemia D and periodic fever syndrome; MKD, mevalonate kinase deficiency; NOD2, Nucleotidebinding oligomerization domain containing 2.

### Comparison of Cytokine Profile of SAIDs with DM

Both SAIDs and DM share similar cytokine profiles. Inflammasome and cytokines like interleukin (IL)-1β, IL-18, and IL-6 play an important role in certain SAIDs.^[25]^ Of these cytokines, IL-18 in particular are increased in DM/ADM patients with ILD.^[26]^ For example, in a study of serum cytokine levels of 22 DM/PM patients and 24 controls, IL-1β and IL-18 levels were significantly higher in patients than controls. ^[27]^ IL-18 blockade may improve DM skin lesions.^[28]^ The NLRP3 inflammasome is implicated in the pathogenesis of DM. ^[29]^ More recently in an NIH study, distinct interferon signatures and cytokine patterns defined additional SAIDs, including two patients with anti-MDA5 positive juvenile DM.^[30]^

## Discussion

Genetic studies of DM have been conducted, including human leukocyte antigen (HLA), cytokine/lymphocyte signaling alleles and epigenetic modification.^[8]^ HLA associations with different MSAs have been reported in some antibody positive subtypes. ^[31]^ Genome-wide association studies (GWASs) have revealed myositis-related genetic loci. An earlier study showed three genes including phospholipase C-like 1, B lymphoid tyrosine kinase and chemokine were associated with DM.^[32]^ Recently, a large GWAS in the Caucasian population with systemic autoimmune diseases ^[33,34]^ identified 26 shared genome-wide significant loci, 22 of which were related to myositis.^[35]^ Nevertheless, the previous genetic studies of DM only used a limited number of single nucleotide polymorphisms (SNPs) to focus on HLA- and immune disease-associated genes for pathogenic mutations. To date, we have not seen any genetic studies to focus on SAID-associated gene mutations in DM or ADM patients. Over the last decade, researchers have searched for rare genetic mutations for monogenic diseases based on the traditional binary classification of human genetic diseases as monogenic and polygenic. However, there are thousands of genetic variants identified for established and emergent diseases. Many variants identified are in the “grey area” between monogenic and polygenic^[36]^ and in fact, some low frequency variants (minor allele frequency 1% to 5%) are linked to SAIDs, such as CAPS, TRAPS, NLRP12-AID and YAOS. ^[14,20]^ To meet the growing need and to supplement the traditional classification, we recently proposed and defined “Genetically Transitional Diseases (GTD) ”, as a new concept in genomic medicine to denote a disease or disease status between monogenic and polygenic diseases. In these diseases a mutation is necessary, but not sufficient to cause disease.^[37]^ We believe that further genetic profiling will be useful in the context of an autoinflammatory disease gene panel to cover both high and low penetrance variants. This may be necessary to identify genetic variants associated with SAIDs in a subgroup of DM or ADM patients.

The central hypothesis of mechanisms underlying SAIDs is abnormal innate immune response. Mechanisms in SAIDs are complex and heterogenous, depending on individual disease categories. For example, for hereditary recurrent fever syndromes and NOD-like receptor (NLR) associated SAIDs (FMF, CAPS, NLRP12-AID), genetic variations, inflammasomes and aberrant cytokines such as IL-1β, IL-8 and IL-18 contribute to these diseases.^[3]^ NOD2-associated diseases, such as Crohn’s disease, Blau syndrome and YAOS are associated with NOD2 mutations that result in abnormal NOD2 activation of RIP2, NF-κB, MAPK, Caspase-1 pathways, as well as cytokine production like IL-1, IL-8 and IL-6.^[38]^ Several new complex clinical autoinflammatory syndromes include systemic inflammation, panniculitis, and myositis due to proteasome defects (PRAAS/CANDLE), vasculopathy, vasculitis, and interstitial lung disease with STING hyperactivity (SAVI), and subacute encephalomyelitis with cerebral calcifications and white matter disease due to cytosolic nucleotide dysregulation (AGS). These diseases are caused by mutations leading to chronic type I interferon (IFN) production as inflammatory mediators that cause autoinflammatory phenotypes. ^[39]^

Interestingly, there are reports to link inflammasome with DM. For example, glycolysis promotes skeletal muscle cell pyroptosis by activating the NLRP3 inflammasome in DM.^[29]^ Since autoantibodies against anti-TIF-1-γ, a member of the tripartite motif (TRIM) proteins, has a strong association with DM related malignancy, certain TRIM member genes are found to be upregulated. ^[40]^ TRIM proteins have been proposed to contribute to the development and pathology of autoimmune and autoinflammatory conditions.^[41]^ Aberrant type I IFN responses are involved in DM. Retrotransposons and IFNs are upregulated in DM patient samples different from SLE, as a characteristic, compared to healthy controls.^[42]^ Type I IFN responses are significantly enhanced in cells from juvenile DM. ^[43]^ These data also support the similarities between SAIDs and DM.

**Figure 2:**
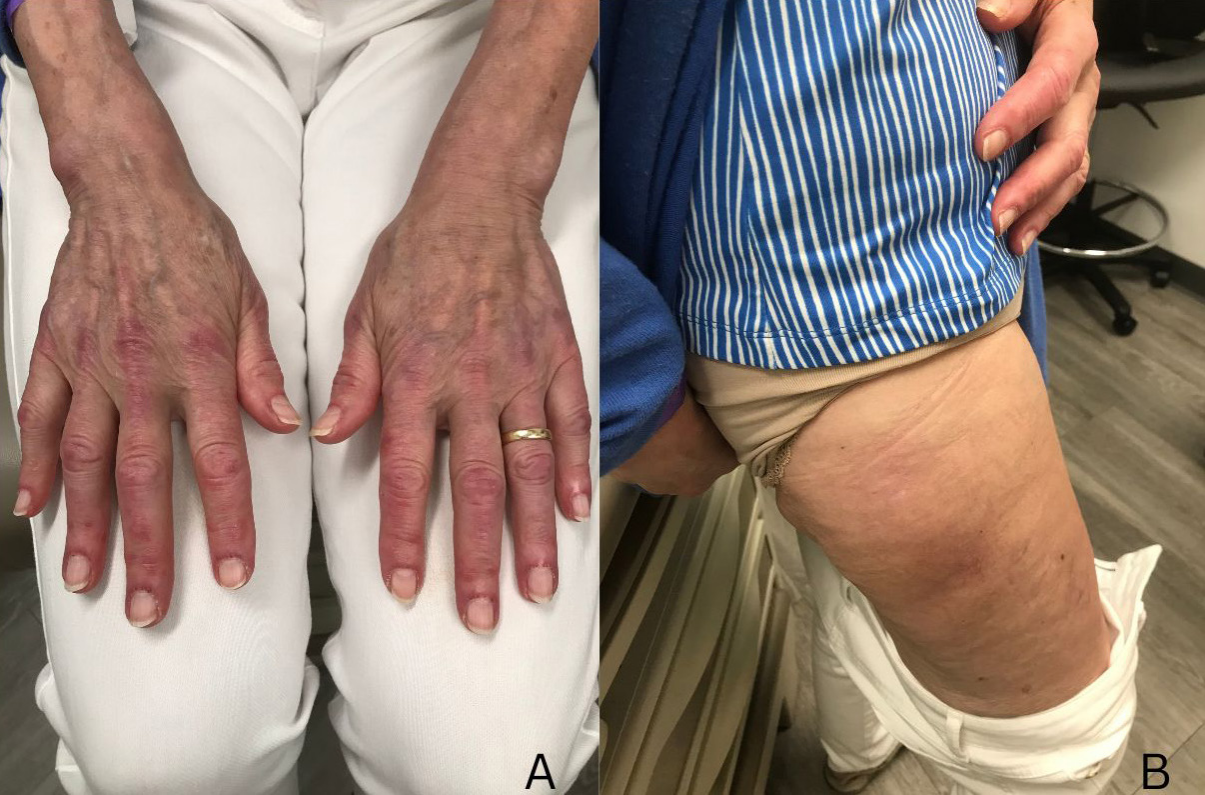
Gottron papules and Holster sign in a patient with Yao syndrome, who carries the NOD2 mutation, p. Pro668Leu.

Currently, therapeutic regimens differ between DM and SAIDs. High-dose glucocorticoids in combination with immunosuppressive agents, such as cyclosporine, tacrolimus, azathioprine, rituximab, or cyclophosphamide are commonly used in patients with DM; ADM can be resistant to immunosuppressive agents with a poor outcome. In contrast, biologics, such as IL-1 inhibitors, are frequently efficacious for patients with SAIDs.^[44]^ Interestingly, there was a case report of refractory DM patient who responded well to anakinra, an IL-1 inhibitor.^[45]^ In a clinical trial of anakinra in 15 inflammatory myositis patients, 3/4 DM patients showed symptomatic improvement. ^[46]^ Other cytokine inhibitors have been under clinical trials as well.^[47]^ Herein, we describe the case of 70-year-old Caucasian female, who carried a diagnosis of DM for a decade based on intermittent facial rash, Gottron sign, and Holster sign (Figure 2), as well as myalgia without elevated muscle enzymes or autoantibodies. Electromyography was unremarkable and a skin biopsy showed a sparse superficial perivascular cell infiltrate composed of predominantly lymphocytes. Genetic testing using an autoinflammatory gene panel identified a heterozygous NOD2 mutation, Pro668Leu (minor allele frequency 0.045% in Genome Aggregation Database [gnomAD]). With periodic occurrence of disease, lack of characteristic histopathologic finding for DM, identification of the rare NOD2 mutation and good response to small doses of prednisone (5 mg daily), Yao syndrome was diagnosed.^[13]^ Taken together, a better understanding of the potential role of autoinflammatory components in DM could form a foundation for genetic testing for SAIDs and for a possible trial of IL-1 inhibitors in selected DM patient population. DM, especially ADM, in the absence of autoantibodies may be on the spectrum of autoinflammatory diseases. Further study of these patients is warranted.

## Conclusion

In summary, both DM and SAIDs share certain clinical phenotypes, cytokine profile, and pathogenic mechanisms, thus they can mimic one another, particularly in the absence of autoantibodies. In this setting, it would be reasonable to refer patients to subspecialists for molecular testing, as some patients with clinically diagnosed DM may harbor SAID-associated gene variants. These patients might benefit from using IL-1/IL-6 inhibitors for SAIDs. We believe this report will increase awareness of SAIDs and their similarities to DM among physicians.
